# Acute Primary Pyelitis—Coli Bacilluria—in Children

**Published:** 1910-11-12

**Authors:** 


### Diseases of Children.

### ACUTE PRIMARY PYELITIS—COLI BACILLURIA—IN CHILDREN.

The recognition of acute primary pyelitis as a definite morbid entity with characteristic symptoms and ætiology is of comparatively recent date. Escherich, Trumpp, and Finklestein were the first observers to term the condition "coli-cystitis," and found that it was confined to female infants, and that the urine in these cases contains pure cultures of the common colon bacillus. Recent observers have shown that the bladder is generally unaffected, and that the morbid condition is confined chiefly to the pelvis of the kidney, but also to the upper urinary passages. Nearly all the cases recorded have occurred in children under the age of two years. Numerous and admirable cases have been described by Holt, Thompson, and Lippe. Fifteen cases recorded by Thompson were all below the age of two, and the same also applies to a series of six cases by Lippe. Primary pyelitis is much more common in female children than it is in males. It is closely analogous to the acute pyelonephritis of pregnancy.

### Onset and Course.

It is not necessary to go into full details, but it may be said that the child, who is usually a female, appears acutely ill and in great distress. The temperature is considerably raised, reaching as high as 102° F. to 105° F., or even higher. On careful examination of the patient there is very seldom any tenderness, any physical signs of disease of the heart, lungs, or abdominal organs, and upon inquiry it will often be ascertained that the onset of the illness was sudden, and in many instances the hour of the commencement of the ailment has been noted by the mother. Convulsions may occur in a few cases, and rigors have also been noted, and when present are a symptom of great diagnostic value, for rigors are rarely present in other acute affections of early childhood. Marked restlessness is often present, and, as may be expected owing to the high temperature of the body, other nervous signs, such as twitching of the face or limbs, or even general convulsions with loss of consciousness may occur. A case has been recorded of a female infant, aged 11½ months, in whom the nervous symptoms were so severe that the diagnosis of tubercular meningitis was made; the child made a rapid recovery under treatment. The tongue is usually clean and the action of the bowels generally normal or costive, but in some cases a temporary attack of diarrhœa has preceded the onset of the affection. The urine is clear or slightly turbid, and strongly acid in reaction; pus is always present in slight quantities, and *can only be detected by microscopical examination*, and then often only a few pus corpuscles are visible in the fluid under the high power of the microscope. Albumin may or may not be detected on examination, and when present it is only in small quantity. When a bacteriological examination of the urine is made it has often been found to reveal practically a pure culture of bacillus coli communis. If a case is not treated at once the temperature assumes an intermittent or remittent type, and may continue for a long period, the child probably becoming wasted, anæmic, and weak.

It is very seldom that fatal cases occur in this affection, and this is probably due to the fact that, once the condition is recognised, the treatment is most satisfactory. Fatal cases may occur, however, and may be ascribed to other conditions, such as typhoid fever for instance.

Those who have carefully observed the clinical history of cases of acute pyelitis are agreed that it is an acute infective condition of the urinary tract, and the presence in the urine of the bacillus coli points to that organism being the chief cause of the trouble. The theory has generally been accepted that pyelitis is due to an ascending infection, the bacilli reaching the pelvis of the kidney by way of the lower urinary tract; but it has been stated that the mode of infection in boys in most cases is transparietal, and in a fair proportion in girls, whilst in both sexes it is occasionally hæmatogenous; in girls the infection is usually through the urethra. The shortness of the female urethra and the age at which this affection occurs chiefly explains why this affection is mostly confined to female infants.

### Diagnosis.

The diagnosis presents no difficulty, but it can only be made by a microscopical examination of the urine. It is advisable, therefore, to make a routine examination of the urine in all cases of young children who are suffering from a pyrexia of obscure origin.

A secondary pyelitis resulting from the formation of calculi in the renal pelvis, or when due to the extension of tuberculosis from the kidney or bladder, is hardly likely to be confused with the primary condition, for in neither of these conditions is there a high degree of pyrexia with an acute and sudden onset. It should be added, however, that pyelitis may be a complication or sequela of infantile scurvy, and in such cases the patient's temperature will be considerably raised.

### Treatment.

The treatment of pyelitis consists for the most part in rendering the urine neutral or alkaline by the administration of citrate of potash, which must be continued for some time after the temperature has become normal, otherwise a relapse may occur. Urotropine has not given uniformly favourable results, acetate of potash and digitalis proving more beneficial.

In a case of pyelitis recorded by Dr. G. B. Sweet, of Sydney, the patient was a healthy little girl aged one year and nine months, who was taken suddenly ill with a feverish attack; her temperature rose to 103° F., she was very thirsty, and her mother had noticed that micturition was frequent but painless; the tongue was clean and the action of the bowels normal. Nothing abnormal could be made out on examination; she was sent to the hospital, and the same evening the temperature rose to 104° F. Citrate of potash was ordered in 5-grain doses thrice daily. A Widal's reaction of her blood was taken and found to be negative. Examination of the urine showed that it was acid in reaction and contained a trace of albumen and numerous pus cells. The dose of citrate of potash was then increased to six grains every four hours. Five days later the temperature reached normal, and there was no further rise. Her general condition then steadily improved, and she gained a pound in weight during her last week's stay in hospital.

### Another Typical Case.

In another case the patient was a girl aged six years, stated to be suffering from typhoid fever. She had been ill for five weeks before admission to the hospital with headache, fever, and drowsiness. The onset of the illness was quite sudden, the patient complaining of feeling ill, with pain in her back, on going to school one morning; this became so bad that she had to return home. She was anæmic when admitted to the hospital, and greatly wasted; her tongue was covered with a thick coating of white fur; the bowels were constipated, and sudamina were present on the surface of the chest and abdomen, but there was no other cutaneous eruption; the abdomen was neither distended nor tender, and the spleen was not palpable; the temperature was 103° F., and the urine had a specific gravity of 1008; it was acid in reaction, and contained no albumen and no sugar. A Widal reaction taken on the day following admission was negative. Two days after her admission the patient complained of abdominal pain, which was subsequently found to be due to a distended bladder. A catheter was passed and 10 oz. of turbid urine were withdrawn, and upon examination it was found to be strongly acid in reaction and to contain numerous pus cells. Citrate of potash, 90 grains daily, was ordered, and two days later the temperature, which previously had been running as high as 104° and 105°, became normal, and remained so for twelve days. The citrate of potash was discontinued, with the result that the temperature rapidly rose to 104°; the following day the mixture was therefore resumed, and the temperature again fell to normal. A bacteriological examination of the urine showed the latter to contain a pure culture of bacillus coli communis. The treatment so far appeared to be very satisfactory, but later three relapses occurred, each lasting from between two to four days. During each relapse the temperature rose, an erythematous rash appeared on the chest and abdomen, and the patient became very drowsy. It was afterwards discovered that the probable cause for these relapses was due to the fact that the nightly doses of the drug had been omitted. The citrate of potash was again continued night and day, and the patient became fat and strong, but the pus cells in the urine took some weeks to eradicate.

### A Third Case.

In a third case the patient was a female child aged two years, who was admitted to the hospital suffering from convulsions, which were attributed to the child having eaten pork sausage some hours before being taken ill. When brought to the hospital she was in a very collapsed condition, the face somewhat cyanosed, the abdomen distended and tender, and the temperature 99° F. On the following morning the temperature rose to 101° F., and the same evening reached 103° F.; by the following evening the temperature was 105° F. On the fourth day after admission a microscopical examination of the urine was made, and it was found to contain pus cells in the centrifuged deposit. Potassium citrate, grains iv., were ordered every two hours. The temperature remained at 103° for the next three days, and then began to fall, reaching normal on the sixth day after the commencement of the treatment. A bacteriological examination of the urine revealed pure cultures of the bacillus coli communis. The child, who had become wasted and anæmic, rapidly improved under the above treatment, and the pus cells gradually disappeared from the urine.

### A Fourth Case.

The last case recorded by Dr. G. B. Sweet appears to present some unusual features, the patient being a girl aged five years, who was admitted to the hospital stated to be suffering from typhoid fever. Eight days previous to admission she suddenly complained of pain in her right lumbar region, which disappeared after a short time; the same night the child became feverish and restless, and the following morning had a rigor, and later in the day had a convulsion. The feverish condition of the patient continued, and she was seen by a doctor, who diagnosed typhoid. On admission the temperature was 102.4, the tongue was clean, and the bowels constipated; the urine was of specific gravity 1020, gave an acid reaction, and contained no albumin, and a Widal's reaction was negative. Five days after admission a microscopical examination of the urine was made, and pus cells were found to be present; the following day a bacteriological examination showed the presence of bacillus coli communis in enormous numbers. The temperature fell to normal after the administration of potassium citrate in 6-grain doses, and the patient was discharged apparently quite well.

### Summary.

From the above recorded cases it will be seen that the chief characteristics of pyelitis are: the sudden onset, the high range of the bodily temperature, the general absence of physical signs of disease, and the satisfactory effects of treatment. Case 2 is particularly interesting on account of the age at which the condition occurred, and of the long febrile period, lasting nearly six weeks, before the diagnosis was confirmed, and the occurrence of several relapses due to the stoppage of citrate of potash and probably insufficient drug treatment.

